# Generation of a vector suite for protein solubility screening

**DOI:** 10.3389/fmicb.2014.00067

**Published:** 2014-02-25

**Authors:** Agustín Correa, Claudia Ortega, Gonzalo Obal, Pedro Alzari, Renaud Vincentelli, Pablo Oppezzo

**Affiliations:** ^1^Recombinant Protein Unit, Institut Pasteur de MontevideoMontevideo, Uruguay; ^2^Protein Biophysics Unit, Institut Pasteur de MontevideoMontevideo, Uruguay; ^3^Unité de Microbiologie Structurale, Institut Pasteur, ParisFrance; ^4^Centre National de la Recherche Scientifique, Aix-Marseille UniversitéCNRS UMR7257, AFMB, Marseille, France

**Keywords:** recombinant proteins, solubility, expression, vector, cloning, high-throughput

## Abstract

Recombinant protein expression has become an invaluable tool for academic and biotechnological projects. With the use of high-throughput screening technologies for soluble protein production, uncountable target proteins have been produced in a soluble and homogeneous state enabling the realization of further studies. Evaluation of hundreds conditions requires the use of high-throughput cloning and screening methods. Here we describe a new versatile vector suite dedicated to the expression improvement of recombinant proteins (RP) with solubility problems. This vector suite allows the parallel cloning of the same PCR product into the 12 different expression vectors evaluating protein expression under different promoter strength, different fusion tags as well as different solubility enhancer proteins. Additionally, we propose the use of a new fusion protein which appears to be a useful solubility enhancer. Above all we propose in this work an economic and useful vector suite to fast track the solubility of different RP. We also propose a new solubility enhancer protein that can be included in the evaluation of the expression of RP that are insoluble in classical expression conditions.

## INTRODUCTION

Recombinant protein production has become a routine practice in many laboratories from academic to industrial fields. Several hosts are available for protein production among them, *Escherichia coli* has been by far the most widely used. Some advantages of this host is the low cost, infrastructure of implementation, easy handling, high yield production, and an ever increasing set of tools and genetic information useful for the expression of challenging targets. Despite its importance and utility, recombinant proteins (RP) not always are produced in a soluble and homogeneous state. For these “difficult to express” proteins, several approaches have been developed in order to overcome the problems associated with insolubility. Some parameters that can affect protein expression are: induction temperature, promoter strength, use of specific *E. coli* strains, co-expression of molecular chaperones or biological partners and the use of different solubility enhancer or fusion proteins (Correa and Oppezzo, 2011). In the last decade, the advent of high-throughput screening methods have facilitated the evaluation of hundreds of conditions generated from the combination of the mentioned parameters in order to find one that gives a soluble protein (Vincentelli et al., 2011; Vincentelli and Romier, 2013). However, to exploit all these variables it is necessary to have a method for cloning the target gene in many different vectors in a fast and simple manner. Several techniques were recently generated to facilitate the cloning of target genes in a parallel way, in which the same insert can be introduced into different expression vectors simultaneously. Among these methods are the Gateway technology [Invitrogen, (Esposito et al., 2009)], In-Fusion technology, [Clontech, (Berrow et al., 2007)], Ligase Independent Cloning, (Aslanidis and de Jong, 1990), and Restriction Free Cloning, [RF cloning, (Unger et al., 2010)]. With these methodologies, the use of restriction endonucleases is avoided, so no special sequence requirements are necessary enabling the development of high-throughput technologies for molecular cloning (Cabrita et al., 2006; Berrow et al., 2007; Curiel et al., 2010; Unger et al., 2010; Luna-Vargas et al., 2011).

In this work, we have modified two commonly used commercial vectors (pET32a and pQE80L, T7 and T5 promoters respectively) for *E. coli* protein expression. We generated 12 different vectors introducing the same sequence at the insertion site, and important features for protein purification like N-terminal (His)_6_ tag (Murphy and Doyle, 2005), TEV cleavage site, and C-terminal StrepTag II (Schmidt and Skerra, 2007), in order to set up a high-throughput cloning and purification protocol. The cloning strategy used for the development of the vectors as well as for cloning the target genes on the entire suite is based in the “RF cloning methodology” (Unger et al., 2010). The data reported here, describe the application of an easy methodology to clone any target in 12 different vectors with only two primers. In order to evaluate and find a condition for soluble protein expression, different promoters and solubility enhancer fusion proteins were included in these vectors. Concerning protein solubility enhancers, the target gene can be fused as a C-terminal partner with maltose binding protein (MBP; Kapust and Waugh, 1999), thioredoxin A (Trx; LaVallie et al., 2000), small ubiquitin-like modifier protein (SUMO; Marblestone et al., 2006), disulfide bond isomerase C (DsbC; Nozach et al., 2013), and Histag alone in a T5 or T7 promoter context.

Finally, we propose a new fusion protein which appears to be an efficient solubility enhancer for the RP with previous solubility problems and is included in the vector suite. This solubility enhancer corresponds to a truncated construct of the endoglucanase CelD (CelDnc) from *Clostridium thermocellum*. This is a thermostable protein, highly expressed in *E. coli* system and more interestingly, this molecule maintains a full activity even in the presence of 8M Urea implying a very high stability of its native structure (Chaffotte et al., 1992). All these characteristics make CelDnc a good candidate to study the solubility enhancing properties when fused a target protein. As a proof of concept, we fused to CelDnc the decaprenylphosphoryl-β-D-ribofuranose-2′-epimerase (DprE1) protein from *Micobacterium smegmatis* (Neres et al., 2012) a difficult protein to express in *E. coli* (<0.4 mg/l) and we successfully improved this expression obtaining high yields of soluble and functional monomeric protein.

In summary, here we illustrate how to generate in any laboratory an economic and useful vector suite to fast track the solubility of different RP targets and we propose a new solubility enhancer protein that can be included in the evaluation of the expression of RP that are insoluble in classical expression conditions.

## RESULTS

### CONSTRUCTION OF A NEW VECTOR SUITE

**FIGURE 1:**
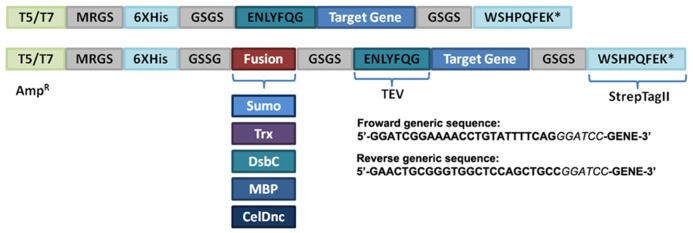
**Schematic representation of vector suite.** The generic module with the different characteristics is shown. T5/T7, promoter type; Amp^R^, ampi-cillin resistance; tobacco etch virus protease recognition site (TEV), 6xHis, His-Tag; Sumo, Trx, DsbC, MBP, and CelDnc are the different fusion proteins. The generic sequences to add to the forward and reverse primers are indicated, italic letters correspond to the *Bam*HI restriction site. * represent a stop codon.

Aiming to achieve a fast and economical way to evaluate the solubility of RP, we selected two commonly used expression vectors pQE-80L (Qiagen) and pET-32a (Novagen) as the starter plasmids for the suite generation thus giving rise to T5 or T7 based vectors. In order to provide a parallel cloning of the target gene and an easy protein purification method, all the generated vectors contain the same insertion site and antibiotic resistance (ampicillin), an N-terminus His-Tag with the tobacco etch virus (TEV) recognition site and a C-terminus strep-Tag II (**Figure 1**; **Table 1**). In addition, we introduced several solubility enhancing proteins including MBP, Trx, DsbC, SUMO, and CelDnc, in combination with the two promoters (T5 or T7). An extra serine residue was added after the TEV site to decrease steric effects and improve cleavage. This can be avoided by not including it in the forward primer. This extra codon also generates a *Bam*HI site at the beginning of the gene so it can be useful for analysis of clones or to do a restriction based method if preferred (**Figure 1**).

**Table 1:** Primer list for vector generation, cloning, and sequencing.

Primer	Sequence 5′–3′	Characteristics
CelDwtNFor	GGATCGGAAAACCTGTATTTTCAGGGATCCATGACCATGATTACGAATTCCCGG	Cloning of CelDwt
CelDwtCRev	GCTGCAGGTCGACGCCAAGATCCTTTTTTATATTGGTAATTTCTCGATTACCCT	
CelDtruncNFor	GGATCGGAAAACCTGTATTTTCAGGGATCCTCGGGATTGATTGAGACCAAAGTG	Cloning of CelDnc
CelDtruncCRev	GCTGCAGGTCGACGCCAAGATCCTTTTTTAAGCAGAATTATAGTTGACAAATCCGG	
QE3790For	GGATCGGAAAACCTGTATTTTCAGGGATCCATGGGCGCGGTACCCTCACTGACG	Cloning of Rv3790
QE3790Rev	GCTGCAGGTCGACGCCAAGATCCTTTTTTAGAGCAGTTGCAGGCGCCTGGCCATG	
CelDInsFor	ATGAGAGGATCGCATCACCATCACCATCACGGATCTTCGGGATTGATTGAGACCAAAGTGTC	Insertion of CelDnc as a fusion partner
CelDInsRev	GGATCCCTGAAAATACAGGTTTTCCGATCCGCTACCAGCAGAATTATAGTTGACAAATC	
strepCterFor	TCCGACATGGCCAGGCGCCTGCAACTGCTCGGATCCGGCAGCTGGAGCCACCCGCAGTTC	Insterion of a C-terminus strepTagII
strepCterRev	TGGCTGCAGGTCGACGCCAAGATCCTTTTTTACTTTTCGAACTGCGGGTGGCTCCAGCTG	
SumoFor	CATCACCATCACCATCACGGATCTTCGGGAATGTCGGACTCAGAAGTCAATCAAG	Insertion of Sumo as a fusion partner
SumoRev	CTGAAAATACAGGTTTTCCGATCCGCTACCATACGTAGCACCACCAATCTGTTC	
TrxFor	CATCACCATCACCATCACGGATCTTCGGGAATGAGCGATAAAATTATTCACCTG	Insertion of TrxA as a fusion partner
TrxRev	CTGAAAATACAGGTTTTCCGATCCGCTACCGGCCAGGTTAGCGTCGAGGAACTC	
MBPFor	CATCACCATCACCATCACGGATCTTCGGGAATGAAAACTGAAGAAGGTAAACTG	Insertion of MBP as a fusion partner
MBPRev	CTGAAAATACAGGTTTTCCGATCCGCTACCATTAGTCTGCGCGTCTTTCAGGGC	
DsbCFor	CATCACCATCACCATCACGGATCTTCGGGAGATGACGCGGCAATTCAACAAACGTTAGCC	Insertion of DsbC as a fusion partner
DsbCRev	CTGAAAATACAGGTTTTCCGATCCGCTACCTTTACCGCTGGTCATTTTTTGGTGTTCGTC	
T5T7For	TTTGTTTAACTTTAAGAAGGAGATATACATATGAGAGGATCGCATCACCATCACCATCAC	Transfer of the entire cassette to pET32a vector
T5T7Rev	CAGTGGTGGTGGTGGTGGTGCTCGAGTGCGGCTTGGCTGCAGGTCGACGC	
GFPFor	GGATCGGAAAACCTGTATTTTCAGGGATCCAGCAAAGGAGGAGAACTTTTC	GFP cloning
GFPRev	GAACTGCGGGTGGCTCCAGCTGCCGGATCCTCAAAGCTTTTTGTAGAGCTCATC	
MPK4For	GGATCGGAAAACCTGTATTTTCAGGGATCCATGGCTCAACTCGTCCCTTTAGC	MPK4 cloning
MPK4Rev	GAACTGCGGGTGGCTCCAGCTGCCGGATCCCTATTCGTTCAATTGTGAATGGG	
MBPSeqFor	CGCTGGCGCGAAAGCGGGTC	MBP sequencing
CelDSeqFor	GTGCCCTGGAGCAGTGCCGC	CelD sequencing

### VALIDATION OF THE NEW VECTOR SUITE

**FIGURE 2:**
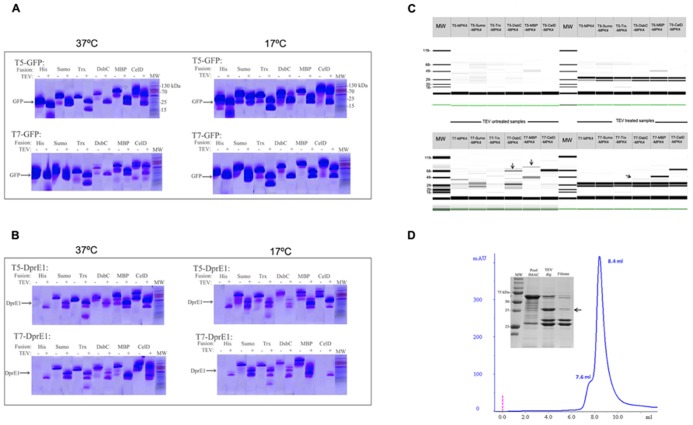
**Protein production screening in the vector suite**. Panels **(A,B)** corresponds to the E-PAGE 96 acrylamide gels for the expression screening of GFP and DprE1, respectively. The incubation with TEV protease for fusion cleavage is indicated with a +sign over the corresponding lines. Cleaved target protein at the expected molecular weight (MW) is depicted. Additionally, induction temperatures are indicated over each panel. **(C)** Expression screening for MPK4 at 17°C using a Labchip GX II (Caliper, USA) microfluidic detection system. Arrows indicate the presence of a band with the expected molecular weight. Construct names are provided over each gel line. Solubility improvement with vector suite is indicated by arrows. **(D)** Analytical size exclusion chromatography (SEC) of the IMAC purified fraction of DsbC-MPK4. Peaks at 7.6 and 8.4 ml correspond to the exclusion volume and the 600 kDa decameric form of DsbC-MPK4, respectively. The 12% SDS-PAGE shows the fusion protein obtained by IMAC purification and DsbC-MPK4 digested by TEV protease. The expected molecular weight of MPK4 (41.7 kDa) is indicated by an arrow.

In order to evaluate the expression capabilities and functionality of this new vector suite we selected green fluorescent protein (GFP) as control protein and two “difficult to express” RP such as DprE1 and the MAP kinase 4 from *Leishmania major* (MPK4). All of them were cloned into 12 different vectors and their expression was evaluated. The results showed that all the GFP constructs were produced soluble and at the expected molecular weight. Fractions treated with TEV showed the correct cleavage and release of GFP protein and fusion partner (**Figure 2A**). The construct DsbC-GFP under the control of T7 promoter was the less productive when working at 37°C. This was over-passed when the expression was done at 17°C over night (ON) where an increment of cleaved proteins was obtained in most of the cases (**Figure 2A**).

For the case of DprE1 constructs, we can see that despite a correct growth and induction conditions in the culture, it was not possible to obtain any expression of this RP when fused only to a Histag. In contrast, fusion of DprE1 with MBP, Sumo, Trx, and CelDnc give a good soluble production and only low yields account for the DsbC/DprE1 construct (**Figure 2B**; **Table 2**). Also, there was an effect of the induction temperature and promoter strength in protein expression where DprE1 was expressed with higher yields at 37°C compared to 17°C and with the T5 promoter compared with T7 for most of the cases. Interestingly, our results suggested that DprE1 fused with CelDnc (in the condition T5-37°C) appear to be one of the most overexpressed fused proteins. For the case of DprE1/CelDnc in T7 at 17°C, there was no cell growth. Finally, the treatment with TEV revealed that DprE1 RP fused with all these enhancers remains in a soluble state confirming an important improvement expression after usage of this vector suite (**Figure 2B**).

**Table 2:** Expression screening of DprE1 protein.

	Construct name	Fusion protein	MW DprE1 fusions (kDa)	Yield at 37°C (mg/l)	Yield at 17°C (mg/l)
**T5 promoter**
	pT5-DprE1	Only HisTag	53.7	0.4	0.2
	pT5-Sumo-DprE1	Sumo	65.5	12.3	14.1
	pT5-Trx-DprE1	Trx	65.8	14.8	10.4
	pT5-DsbC-DprE1	DsbC	77.4	6.2	4.7
	pT5-MBP-DprE1	MBP	94.3	15.4	11.3
	pT5-CelD-DprE1	CelDnc	114.8	19.5	12.8
**T7 promoter**
	pT7-DprE1	Only HisTag	53.7	0.1	0.2
	pT7-Sumo-DprE1	Sumo	65.5	11.6	10.1
	pT7-Trx-DprE1	Trx	65.8	12.4	9.8
	pT7-DsbC-DprE1	DsbC	77.4	8.1	3.9
	pT7-MBP-DprE1	MBP	94.3	12.8	15.8
	pT7-CelD-DprE1	CelDnc	114.8	19.2	ND

*
After purification by IMAC, concentration of the entire fusions and yield was determined at 280 nm taking into account the different extinction coefficients. The expected molecular weight as well as construct name and characteristics are indicated.*

Concerning MPK4 our results showed that of the 12 constructs only 2 gave a band at the expected molecular weight. These correspond to the construct pT7-DsbC-MPK4 and pT7-MBP-MPK4 (**Figure 2C**). In both cases TEV protease was able to cleave the fusion but only in the pT7-DsbC-MPK4 constructs**it**was possible to get a soluble protein after cleavage (**Figure 2C**, TEV treatment section). In order to confirm this result and validate our suite vector we proceed to perform a large scale purification with this construct. Our results showed that after protein purification by IMAC it is possible to obtain the DsbC-MPK4 fusion in a soluble manner and with a yield of 6 mg/l (**Figure 2D**). Oligomeric state analysis of the DsbC-MPK4 fusion, revealed that the eluted peak is maintained as a soluble decameric oligomer with an apparent molecular weight of around 650 kDa (**Figure 2D**). This result was verified by dynamic light scattering (data not shown). Despite the fact, a great part of the MPK4 protein precipitate after TEV treatment, an interesting and scalable amount of this protein remains in a soluble form (**Figure 2D**).

Altogether these results, underline the importance of this new vector suite as an improved tool for the soluble expression of DprE1 and MPK4 proteins and suggest that it can be very valuable for the expression of other “difficult to express” RP.

### USE OF ENDOGLUCONASE D VARIANT (CelDnc) FOR THE SOLUBLE EXPRESSION OF DprE1 PROTEIN

**FIGURE 3:**
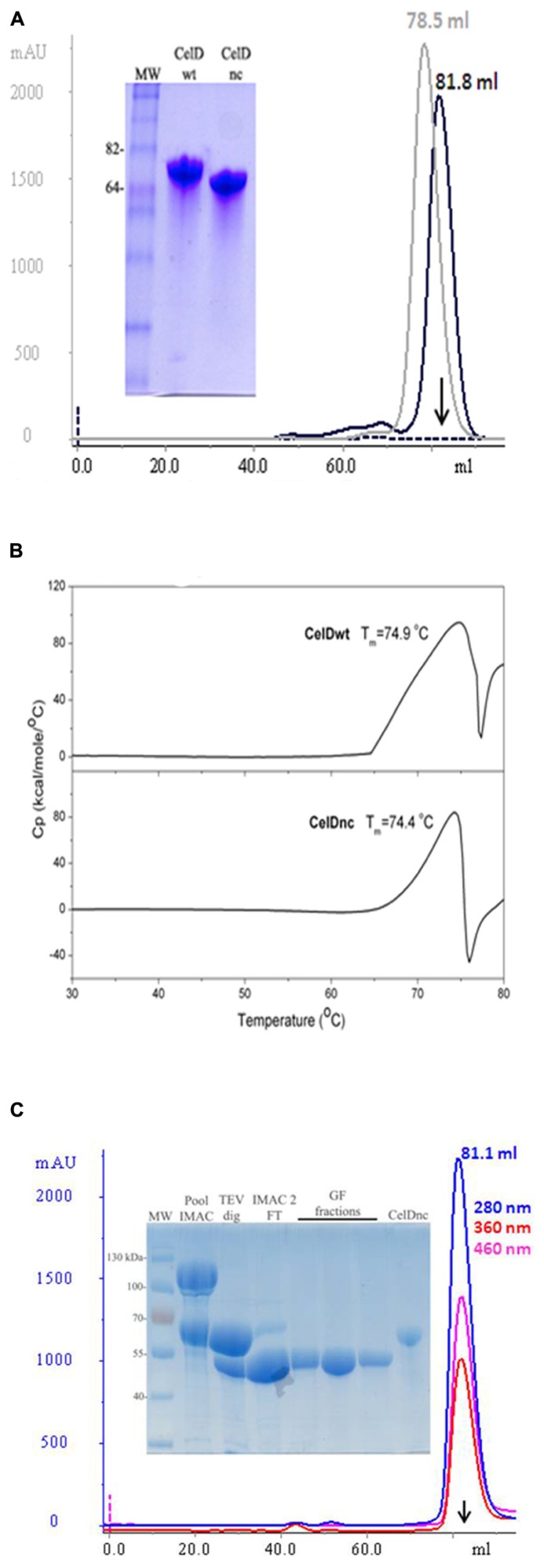
**(A)** Analysis of the purity and monomeric states of CelDwt (gray) and CelDnc (black). SEC was performed in a Superdex 200 16/60 and protein purity evaluated in a 10% SDS-PAGE. **(B)** Differential scanning calorimetry (DSC) curves of CelDwt (top panel) and CelDnc (bottom panel). Determined melting temperature (*T*_m_) is indicated for each case. **(C)** Large scale expression and purification of DprE1. DprE1 was fused to CelD, expressed, and purified by IMAC. After TEV cleavage and second IMAC purification, the monomeric state was confirmed by SEC in a Superdex 200 16/60. FAD binding properties of DprE1 are confirmed by peaks at 360 nm (red) and 460 nm (pink). Purity of DprE1 (53.7 kDa) was evaluated by 12% SDS-PAGE. CelDnc (61.1 kDa) was added as a control. Arrows indicates the retention volume for BSA (66.5 kDa).

After expressing the new construct (CelDnc), we found out that it is expressed at high yields (>400 mg/l) in a soluble monomeric and functional form which in turn maintains thermostable characteristics as the entire version (**Figures 3A,B**). So, we wondered if this extreme solubility and stability could help in the production and folding of other target proteins. In this regard, we fused CelDnc to the N-terminus of the protein DprE1. The results showed that the fusion was successfully produced in a soluble manner and that after TEV treatment and gel filtration purification it remains soluble, monomeric and it was able to retain a FAD binding property as expected for this protein (peaks at 360 and 460 nm; **Figure 3C**). The final yield was of 7 mg/l which corresponds to more than 17 times improvement in soluble protein expression when compared with no fusion (<0.4 mg/l). Moreover, the same experiment done with MBP fusion resulted in a final yield for DprE1 of 2.8 mg/l (data not shown), demonstrating the usefulness of CelDnc as a solubility enhancer of RP.

These results suggest that the construct CelDnc is an interesting new solubility enhancer that could be taken into account for the expression screening of “difficult to express” RP.

## DISCUSSION

Purified and soluble proteins are essential tools in academic, industrial and medical areas. The knowledge of the molecular structure of individual proteins allow addressing important questions about the physiological function of these molecules, so as to know the biochemical and regulatory pathways in which they are implicated. However, a common scenario is that the first attempt for obtaining soluble protein often fails, requiring the optimization of many parameters increasing production costs and time. One of the standard procedures to circumvent this problem is to screen a series of constructs to identify the optimal vector and culture conditions able to produce enough soluble protein. This may also include the expression of the full-length protein, mutated and/or truncated variants, as well as specific domains of RP (Dahlroth et al., 2006; Yumerefendi et al., 2010). Series of fusion partners may also be investigated for their effects on driving enhanced expression or their capacity to capture and purify the target protein quickly with minimal impurities (Young et al., 2012).

In this work, we describe the generation of a vector suite composed of 12 different expression vectors using the RF cloning method. This suite engages the expression of the RP with strong promoters such as T7 or T5, with N-terminus His-tag, a TEV specific cleavage site and a C-terminus StrepTag II as well as different fusion proteins such as Sumo, Trx, DsbC, MBP, and CelDnc. All these vectors contain the same site of insertion in order to enable a parallel cloning for solubility screening and the posterior large scale purification in a simple and general manner (IMAC purification, TEV cleavage and dialysis, 2nd IMAC). The suite is based on the commonly used pET and pQE vectors and presents no major changes in expression or sequencing protocols. The cloning strategy occurs in an insert-sequence independent manner, with the additional advantage that no restriction site or extra aminoacids are added to the N-terminus of the expressed protein after TEV cleavage, apart from the last glycine residue. As purification features we selected the use of the HisTag, because it has demonstrated to be very versatile, cheap and to work well in small and large scale purifications (Schafer et al., 2002; Steen et al., 2006). Additionally, if the stop codon of the target gene is omitted, an additional purification tag, the strepTag II is expressed in the C-terminus of the target protein. This last can be useful if degradation intermediates appear by coupling IMAC purification with StrepTacting purification only a product with an intact N- and C-terminus will be purified. Also the purification via the StrepTag II showed to be very useful for proteins that are expressed in low abundance where usually purification by IMAC gives many contaminants from the host (Magnusdottir et al., 2009). Finally the TEV site was chosen for protein cleavage as it has demonstrated to be very specific, work well at low temperatures and can be produced in the laboratory with high yield reducing production costs (van den Berg et al., 2006). Moreover, it was shown that the last residue of the cleavage site (Gly) can be changed for all the other residues except for proline for an expense in cleavage efficiency, so if a protein with a native N terminus is needed it can be taken into account (Kapust et al., 2002).

The suite was tested with GFP, and we found out that in all cases there were expression and cleavage with TEV demonstrating that all the vectors worked well. By using this suite of vectors the high-throughput screening for soluble expression could be easily achieved manually or automatically as it was demonstrated for the expression of GFP, DprE1 and MPK4.

In order to challenge the vector suite proposed here we selected two “difficult to express” RP like DprE1, and MPK4. For the first protein evaluated (DprE1) the vector suite demonstrated that the expression protein improved when the target protein was fused to Sumo, Trx, DsbC, MBP, and CelDnc solubility enhancer proteins. Among them the best results concerning solubility and quantities of stable protein was achieved when DprE1 was fused to CelDnc and subsequently cleaved by TEV. In the second case, only two out of 12 conditions evaluated were able to express MPK4 in the soluble fraction and only one (pT7-DsbC-MPK4 construct) remains soluble after TEV cleavage. Interestingly, high yield of this fusion construct remained as a decamer before TEV cleavage, so after improving purification protocols (like the use of strepTag II or ion exchange chromatography), the entire fusion can be used for crystallization screenings.

Despite the fact that, many fusion proteins were evaluated, it remains difficult to define a “universal fusion protein.” Different options are commercially available (MBP, GST, Trx, DsbC, NusA, etc), and several groups have found new proteins that can be promising alternatives to obtain a soluble and homogeneous recombinant protein (Chatterjee and Esposito, 2006; DelProposto et al., 2009; Cheng et al., 2010; Song et al., 2011) by fusing the target gene. In this work, we evaluated the use of a novel fusion protein, CelDnc that is thermostable (Tm: 71.4°C) and is expressed in massive amounts in *E coli* system. CelD is an endo-β-glucanase (EC 3.2.1.4) from *C. thermocellum* and is part of the cellulose degrading complex termed cellulosome composed of a large number of individual enzymes (Kataeva et al., 1997).

When this protein was evaluated as a solubility fusion enhancer for DprE1 the results showed an increasing solubility performance for this molecule compared with other classical fusion enhancers like MBP. After expression and IMAC purification was done the CelDnc fusion was soluble in large amounts. Moreover, DprE1 was still soluble, monomeric and presented FAD binding properties even after the proteolitical removal of CelDnc demonstrating the utility of this fusion protein that can be taken into account when solubility screening is performed.

In this work we propose a new vector suite and a new fusion enhancer molecule with chances to improve the solubility of different RP. The vector suite proposed here allows the evaluation of five different fusion proteins or only the HisTag in combination with two different promoters, giving rise to 12 different constructs for a single target gene. Altogether, our results suggest that this expression system could be an interesting tool to improve solubility problems of RP.

Moreover, the screening protocol can be further improved. In the present work we used Rosetta cells for the screening of RP production. Different *E. coli* strains can be evaluated in parallel like the use of strains for disulfide bond formation (Shuffle, New Engalnd Biolabs), reduced mRNA degradation (BL21 Star, Invitrogen) among others. Also, the co-expression of chaperones or molecular partners can be included if they are in a vector compatible with a ColE1 replication origin. By the complementation of such variables with the vector suite, a great number of conditions can be screened, increasing the chances of finding the optimal context for target protein production.

It was shown that the sequence at the translation initiation region (TIR) can have a detrimental effect in protein production due to the generation of secondary structures in the messenger RNA that can hamper the translation by the ribosome complex. In this regard a predictive method was developed for designing synthetic ribosome binding sites (RBS) that can minimize the formation of secondary structures at RNA level, so increasing the translation rate (Salis et al., 2009; Salis, 2011). Because the nucleotide sequence from +1 to +25 is the same in all vectors, a new RBS can be designed and introduced into the entire suite increasing translation rates.

Finally, despite the cloning of target genes into the suite was very efficient, false positives were found in some cases. This can be improved, for example, if a toxic gene like the toxin CcdB of type II toxin-antitoxin system is added at the insertion site.

Despite the fact that, more proteins should be tested in this vector suite and that there is no magic formula able to ensure the solubility of different proteins, this could be a useful and economic model to fast track the soluble expression of the RP.

## MATERIALS AND METHODS

### GENERATION OF THE VECTOR SUITE

For the generation of the vector suite we used a modified version of the pQE80L (Qiagen) as the starter plasmid, that contained a TEV cleavage site after the Histag separated by a GSGS linker (pQE80L-TEV). In a first step we cloned the gene DprE1 into this vector and added the different modules for the vector suite (linkers, strepTag and different fusion proteins) thus generating the T5 series. Then the entire constructs were cloned into the vector pET32a in order to generate the T7 series.

All PCR were done using Phusion polymerase (Finnzymes). For the amplification of the fragments (megaprimer generation) conditions were 30 s at 98°C and 28 cycles of 98°C for 10 s, 59°C for 1 min and 72°C for 1 min with a final extension step at 72°C for 5 min and PCR products were purified by agarose gel. The generated megaprimers contained 30 bp in both ends that overlaps with the insertion site in the destination vectors. The integration into the vectors was done by RF cloning (Unger et al., 2010) and the RF reaction was as follows: 30 s at 98°C and 30 cycles of 98°C for 10 s, 60°C for 1 min and 72°C for 5 min with a final extension step at 72°C for 7 min. For RF reactions 120 ng of megaprimers and 30 ng destination vector were used. 20 μl were digested with 2 μl Fast Digest DpnI (Thermo) for 15 min at 37°C in order to remove parental plasmid, and 5 μl were used to transform 50 μl of competent DH5α *E. coli* cells. Positive clones were confirmed by colony PCR by using Taq polymerase (Invitrogen) with the same primers used for megaprimer generation. Colony PCR was as follows, 95°C for 3 min, 25 cycles of 95°C for 30 s, 60°C for 30 s and 72°C for 2 min followed by a final extension step at 72°C for 5 min. Positive colonies were selected for plasmid extraction and confirmed by sequencing.

The gene for DprE1 was amplified from *M. smegmatis* genomic DNA using the primers QE3790For and QE3790Rev for the generation of the megaprimer (**Table 1**). The product was cloned into the vector pQE80L-TEV by RF cloning to generate the construct pDprE1. The genes coding for CelDwt or the truncated version CelDnc (residues 32–577), were amplified from the plasmid pCT603 (Chaffotte et al., 1992) with the primers CelDwtNFor and CelDwtCRev for CelDwt and primers CelDtruncNFor and CelDtruncCRev for CelDnc (**Table 1**) and cloned by RF in the same vector to generate the constructs pCelD and pCelDnc. The construct pDprE1 was used for the insertion of CelDnc in the 5′ of DprE1 (between the HisTag and the GSGS linker, **Figure 1**). CelDnc was amplified from the pCelDnc construct using primers CelDInsFor and CelDInsRev. The forward primer was designed also to add a GSSG linker to separate the HisTag from the fusion partner generating the construct pCelD-DprE1. The generated constructs (pDprE1 and pCelD-DprE1) were then used to add the last module of the vector, the C-terminal strepTag II. The strepTag II was inserted at the C-terminus separated by a GSGS linker with primers strepCterFor and strepCterRev (**Table 1**) for the generation of the vector pT5-DprE1 (HisTag alone) and pT5-CelD-DprE1 (CelDnc fusion). The primers anneal each other, so they were used without addition of DNA for the generation of the megaprimer. The generated pT5-CelD-DprE1 vector was then used for the insertion and replacement of CelDnc by other fusion partners. In this regard the primers SumoFor and SumoRev; TrxFor, and TrxRev; MBPFor and MBPRev and DsbCFor and DsbCRev were used for the insertion of Sumo, TrxA, MBP, and DsbC, respectively, (**Table 1**). The genes were amplified from *Saccharomyces cerevisiae* for Sumo, pET32a (Novagen) for TrxA, pMAL (New England Biolabs) for MBP; and *E. coli *genome**for DsbC. By this way, the T5 vector series was completed. All 6 vectors were confirmed by sequencing with the QEFor and QERev plasmid primers. For the case of MBP and CelDnc constructs internal primers were also used in order to cover the entire sequence. The last step was to transfer the modules into a T7 context. To do this, we selected the pET32a (Novagen) as a destination vectoramplifying the entire cassette from T5 series (from MRGS-HisTag up to the strepTag II for the different fusions) with the primers T5T7For and T5T7Rev and replacing the expression cassette of the pET32a vector. The generated megaprimers were used for the RF reactions. By this way the vector suite was completed containing the gene DprE1 in all 12 vectors for expression screening.

### CLONING OF GFP AND MPK4 INTO THE SUITE OF VECTORS

*Leishmania major* MPK4 gene was amplified with primers MPK4For and MPK4Rev from a pGem vector containing the gene. GFP was amplified with primers GFPFor and GFPRev from a pET vector containing a GFP variant that is well expressed in *E. coli* (Waldo et al., 1999).

The 12 vectors were added to 12 different PCR tubes, and the amplified products were used as megaprimers for the RF reaction using the HF buffer from Phusion polymerase. After digestion of 20 μl PCR products with 2 μl DpnI, chemical competent cells were transformed with 5 μl RF reaction in a PCR machine with the following program: 30 min at 4°C, 45 s at 42°C, 3 min at 4°C, addition of 100 μl of LB, 1 h at 37°C, and plating of 100 μl in agar plates containing ampicillin. Four colonies for each construct were selected and confirmed by colony PCR and sequenced. After the analysis we found out that in most cases all were positive (or at least three of four were positive) giving a percentage of success of more than 80%.

### EXPRESSION SCREENING OF GFP AND DprE1

Chemocompetent Rosetta-pLysS cells were transformed with 5 μl of purified plasmids as described above and then incubated in a shaker ON at 37°C in 1 ml of LB with chloramphenicol and ampicillin in a 96 × deep-well plate. 100 μl of ON culture were used to inoculate 4 ml of Terrific Broth in 24 × deep-well plates by duplicate. Cultures were incubated at 37°C until D.O._600_ reached 1.0–1.2. At that moment one plate was induced with 1 mM IPTG and left at 37°C for 4 h. The other 24 deep-well was incubated at 17°C for 15 min to cooling it and then induced with 1 mM IPTG ON at the same temperature. After induction time was reached, cells were harvested, resuspended in 1 ml lysis buffer (50 mM Tris pH 8.0; 300 mM NaCl, 10 mM imidazol, 0.5 mg/ml lysozyme) and frozen at -80°C. After thawing cells, 10 units of DNase I and 10 μl of 2M MgSO_4_ were added and incubated with shaking for 20 min at 20°C. Then 200 μl of Nickel beads (Qiagen) equilibrated in binding buffer (50 mM Tris pH 8.0; 300 mM NaCl, 10 mM imidazol) were added to cell extracts and incubated for 15 min at 20°C. Cell extracts were then transferred to a 96×-well filter plate assembled in a vacuum device, and bound protein was washed with 2 ml of binding buffer. An additional wash step was done with 2 ml of binding buffer containing 50 mM imidazol. Elution was done with 160 μl of elution buffer [50 mM Tris pH 8.0; 300 mM NaCl, 500 mM imidazol; for a detailed protocol, see (Saez and Vincentelli, 2013)]. Eluates were divided in two groups for evaluation of uncleaved protein and assessment of TEV cleavage ON at 18°C. Samples were then loaded into an E-PAGE 96 acrylamide gel (Invitrogen).

### EXPRESSION SCREENING OF MPK4

Expression screening and purification of MPK4 constructs was made in a similar way than for GFP and DprE1 but only 17°C of induction was evaluated. Purification steps were the same but the pipeting scheme was done automatically by using a TECAN Freedom EVO®200. Expression analysis was done also automatically by using a Labchip GX II (Caliper, USA) microfluidic detection system.

### LARGE SCALE EXPRESSION AND PURIFICATION OF DsbC-MPK4

DsbC-MPK4 was expressed in Terrific Broth (TB) supplemented with ampicillin and chloramphenicol and induction was done at D.O_600_: 1.2 ON at 17°C with 1 mM IPTG. Pellets were resuspended in lysis buffer and frozen at -80°C. After thawing, the pellets were sonicated and centrifugated at 15.000 × *g*. Soluble fraction was injected in a 1 ml IMAC column (GE Healthcare) equilibrated in binding buffer. Elution was done in a linear gradient of 5–100% B in 10 column volumes (CV) with elution buffer. Purified protein was cleaved with TEV protease in a 1:30 protein:enzyme ratio and dialyzed against cleavage buffer (50 mM Tris pH 8.0; 150 mM NaCl, 1 mM DTT) ON at 8°C. Sample was filtered through 0.22 μm to remove precipitates, and analyzed by SDS-PAGE.

### EXPRESSION AND PURIFICATION OF CelD AND CelDnc

Production of CelD and CelDnc was done in M15pREP4 from the constructs pCelD and pCelDnc, respectively, in 1 l 2YT supplemented with ampicillin and kanamycin, and induced with 1 mM IPTG at D.O. 1.0 ON at 37°C. IMAC was done like for the case of DsbC-MPK4 but using a 5 ml column and only half of the soluble fraction was used. TEV cleavage was done as before and desalted in order to remove imidazole. The reaction was injected in a second IMAC under same conditions as above and the flow through containing the cleaved protein was injected in a Superdex 200 16/60 (GE Healthcare) equilibrated with buffer 40 mM Tris pH 7.7.

### DSC ANALYSIS OF CelD AND CelDnc

Differential scanning calorimetry (DSC) experiments were carried out in PBS, in a VP-DSC instrument (Microcal, Northampton, MA, USA) and data analyzed with the software supplied with the equipment. The temperature was increased at 1°C per minute from 30 to 80°C, and proteins were added at concentration of 1 mg/ml for CelD and CelDnc.

### LARGE SCALE EXPRESSION AND PURIFICATION OF pT5-DprE1, pT5-CelD-DprE1 AND pT5-MBP-DprE1

Induction of p5DprE1, p5CelDnc-DprE1 and p5MBP-DprE1 were done in M15pREP4 with 1 mM IPTG in 1 l 2YT supplemented with ampicillin (100 μg/ml), kanamycin (50 μg/ml) and 15 μM FAD at D.O.: 1.0–1.2 during 4 h at 37°C. Cells were harvested, resuspended in lysis buffer and frozen at -80°C. After thawing the cells, were lysed and protein purified as before. Purified protein was cleaved with TEV in a 1:30 ratio, and dialysed against cleavage buffer. The product was then purified by a second IMAC and injected in a Superdex 200 16/60 equilibrated with buffer 25 mM Tris pH 8.0; 150 mM NaCl.

## Conflict of Interest Statement

The authors declare that the research was conducted in the absence of any commercial or financial relationships that could be construed as a potential conflict of interest.
